# Using artificial agents to nudge outgroup altruism and reduce ingroup favoritism in human-agent interaction

**DOI:** 10.1038/s41598-024-64682-5

**Published:** 2024-07-09

**Authors:** Kevin Igwe, Kevin Durrheim

**Affiliations:** Department of Psychology, Faculty of Humanities, University of Johannesburg, Bunting Road, Auckland Park, Johannesburg, 2092 South Africa

**Keywords:** Ingroup favoritism, Intergroup cooperation, Nudging prosocial behavior, Human-agent interaction, Reputation, Human behaviour, Computer science

## Abstract

Ingroup favoritism and intergroup discrimination can be mutually reinforcing during social interaction, threatening intergroup cooperation and the sustainability of societies. In two studies (N = 880), we investigated whether promoting prosocial outgroup altruism would weaken the ingroup favoritism cycle of influence. Using novel methods of human-agent interaction via a computer-mediated experimental platform, we introduced outgroup altruism by (i) nonadaptive artificial agents with preprogrammed outgroup altruistic behavior (Study 1; N = 400) and (ii) adaptive artificial agents whose altruistic behavior was informed by the prediction of a machine learning algorithm (Study 2; N = 480). A rating task ensured that the observed behavior did not result from the participant’s awareness of the artificial agents. In Study 1, nonadaptive agents prompted ingroup members to withhold cooperation from ingroup agents and reinforced ingroup favoritism among humans. In Study 2, adaptive agents were able to weaken ingroup favoritism over time by maintaining a good reputation with both the ingroup and outgroup members, who perceived agents as being fairer than humans and rated agents as more human than humans. We conclude that a good reputation of the individual exhibiting outgroup altruism is necessary to weaken ingroup favoritism and improve intergroup cooperation. Thus, reputation is important for designing nudge agents.

Group membership influences social behavior, most notably in the phenomenon of ingroup favoritism, which is the tendency for individuals to be more cooperative with ingroup than outgroup members^1^. Fostering intergroup cooperation is necessary for reducing intergroup conflict and competition that threaten global wellbeing^2,3^. However, ingroup favoritism is a ubiquitous and powerful force that is underpinned by deeply ingrained beliefs and expectations about ingroup and outgroup behaviors. We introduced non-human agents into exchange environments to challenge these beliefs and expectations and promote intergroup cooperation.

Research demonstrates that belief about other ingroup and outgroup members’ cooperation shapes intergroup interactions^1,4,5^. Individuals are more inclined to cooperate with their own group because of the belief that ingroup members are more likely to reciprocate such cooperation compared to outgroup members^4,6^. These beliefs reinforce group cohesion but can also reduce cooperation between groups^7,8^, resulting in the increased likelihood of intergroup conflict^1,9–11^.

Recent advances in artificial intelligence have led to artificial agents being used to nudge prosocial behaviors among humans^12,13^. See^14^ for a review. Prosocial behavior is generally associated with two concepts: emotion and altruism^14^. We used novel methods of human-agent interaction over time through a computer-mediated experimental platform to investigate outgroup altruism as a means of altering individuals’ beliefs about intergroup cooperation. Outgroup altruism was introduced via nonadaptive artificial agents whose behavior was preprogrammed to be altruistic (Study 1; N = 400), and adaptive artificial agents whose altruistic behavior was informed by a machine learning algorithm (Study 2; N = 480). The strength of the interventions was measured by the extent to which they affected cooperation with outgroup over ingroup members. Successful nudge agents would be able to build intergroup cooperation by weakening ingroup favoritism. Understanding how to regulate ingroup favoritism can help institutions create policies that promote equitable societies.

## Intergroup cooperation: reciprocity, reputation, and trust as mechanisms

The question of whether people favor ingroup members over outgroup members, i.e., ingroup favoritism, has received much attention^1,15–20^. A great deal of research shows that individuals in various circumstances will discriminate against the outgroup by showing ingroup favoritism in cooperating behavior^1,9,21,22^. Reducing ingroup favoritism is one of the many ways of promoting intergroup cooperation^10,23^. For example, Oyler et al. (2022) outlined various interventions that mitigate intergroup discrimination, including mindfulness intervention, which fosters present-moment attention, promotes a non-judgmental attitude towards outgroup members, and thus weakens ingroup favoritism.

Factors such as perceived intergroup similarity^10^, interdependence^2^, reciprocity, and trust influence whether competition or cooperation occurs between groups^2,24^. For example, interpersonal reciprocity can foster cooperation that can extend beyond group boundaries^3^. Frequent intergroup interactions are important in shifting from ingroup cooperation to more expansive universal cooperation that benefits both the ingroup and outgroup members^3^. Universal cooperation can evolve when the reciprocity of cooperative acts permeates group boundaries^3^. This is particularly relevant when individuals start to reward cooperation, regardless of group membership.

We propose that outgroup favoring agents could elicit reciprocity to effectively challenge and reshape beliefs about ingroup cooperation, promoting more inclusive behavior across diverse groups. Reciprocity norm has been found to increase cooperation and gratitude beyond the benefactor, paying it forward to others^25,26^, permeating group boundaries. Furthermore, increasing the fluidity of group boundaries could foster a broader sense of fairness and cooperation among group members^3^. This fluidity can be facilitated by frequent interactions with outgroup favoring agents, thereby influencing the perception of group boundaries. This could also lead to the perception of shared group membership where outgroup members no longer perceive the agents as an outgroup. Shared group membership is an effective method of reducing intergroup discrimination^27^.

In addition to reciprocity, some research indicates that reputational concerns underpin ingroup favoritism in cooperation^5,28,29^ but see^4^. For example, Wang, et al.^28^ used the Intergroup Parochial and Universal-Cooperation (IPUC) model, which explores varying scenarios of cooperation across groups. They found that reputational concern can lead to different outcomes, depending on the level at which it was formed. At the individual level, concern about personal reputation leads to ingroup favoritism motivated by the desire to maintain a positive reputation in the ingroup^5,29^. Conversely, seeking group reputation leads to intergroup cooperation^28^.

Personal reputation can also be enhanced by adherence to group norms^30^. We define ingroup norms as shared expectations and rules that guide ingroup members’ behaviors. For example, individuals may seek a positive reputation in the group by cooperating only with the ingroup members. Although a recent study^31^ found that reputational concern, the fear of negative evaluation or punishment by ingroup members was not associated with ingroup cooperation. Others, for example^30^ indicated that violating group norms can lead to indirect or direct forms of punishment, like confrontation. Indirect forms of punishment, like gossip^32^, social exclusion^30^, and even withdrawing cooperation^33^ are more commonly used when the perceived risk of retaliation is high^30^. This underlines the importance of balancing individual and group reputations to maintain intergroup cooperation without group disintegration.

Whereas cooperation has been discussed in situations where the benefits of cooperation **b** exceed the cost **c**, that is, ${\varvec{b}}/{\varvec{c}} > 1$ ^34^, a recent study has demonstrated that trust also drives cooperation in trivial giving, where direct or substantial material benefits are not immediately evident^35^. Trivial giving serves as a pure signal of the willingness to cooperate in future interactions, and, people are more likely to engage in trivial giving when they anticipate future interactions with the same partners.^35^. This suggests that the value of cooperation may extend beyond material interests to building trusting relationships and reputation. Consequently, withholding cooperation can be used as a means of expressing distrust and disrepute in trivial giving. In the context of trivial giving, we explore whether outgroup favoring agents can reduce ingroup favoritism by altering individuals’ expectations about others’ ingroup cooperative behavior. To avoid agents being perceived as norm violators, we employ the concept of nudging.

## Nudging cooperation

Nudging has been established as a successful mechanism for promoting behavior without forcing people to accept the behavior^36^, thus minimizing the possibility of a negative experience. Caraban et al.^37^ described 23 ways to nudge, of which we focus on two, namely, reciprocity and social comparisons. Caraban et al. (2019) categorized reciprocity as an automatic process, reasoning that the reciprocation motive is a deeply engrained unconscious response. In contrast, they depicted nudges that enable social comparisons as reflective and transparent^37^, producing change by the process of observation and imitation.

We applied the idea of reciprocity nudging in Caraban et al.^37^ in an intergroup context, and consider how outgroup favoring agents might nudge a reduction of ingroup favoritism by promoting reciprocation and social comparisons in a small group exchange environment. Our outgroup favoring agents serve as intergroup cooperation exemplars that can stimulate reciprocation and nudge ingroup members to observe and reflect on interactions between ingroup and outgroup members. We anticipate that human participants will imitate these acts of outgroup altruism, and will be rewarded via reciprocation. Nudging outgroup altruism can produce intergroup cooperation, but it may have unintended ingroup consequences, undermining the reputation of individuals now viewed as ingroup traitors or norm violators^1,5^, and leading to the withdrawal of cooperation by ingroup members in subsequent interactions^33^.

## The present studies

In two studies, we aimed at nudging outgroup cooperation. We employed two nudge mechanisms: (1) invoking the feeling of reciprocity and (2) imitation by social comparisons. Our assumption was that invoking the feeling of reciprocity would encourage outgroup altruism among outgroup members, while social comparisons would promote outgroup altruism among ingroup members via imitation.

To assess the effectiveness of the intervention in both studies, we evaluated the extent to which outgroup favoring agents reduced ingroup favoritism among human participants, the extent to which the agents gained a bad reputation, and the extent to which ingroup humans withdrew cooperation from ingroup agents. In our study, withdrawing cooperation refers to ingroup members withholding token allocations from ingroup agents that favor outgroups. Fairness is an important component of reputation on which people base their social behavior^38^. Thus, reputation was measured through a fairness rating task. The concepts used in our studies are outlined in Table 1.

**Table 1:** The concept employed, from nudging (top) to validation of the nudge effects (bottom).

Concept	Clarification
Nudger	• Nonadaptive agents (study 1) • Adaptive agents (study 2)
Mechanisms	• Invoking the feeling of reciprocity • Social comparisons
Manifestation	• Reciprocity • Imitation
Expected effect	• Reduced ingroup favoritism
Measures	• The ratio of ingroup allocation to the sum of ingroup and outgroup allocation • Mean fairness rating • Ingroup participants’ cooperation with ingroup agents vs other ingroup participants
Manipulation check	• Mean humanness rating

We conducted the experiments in the Virtual Interaction Application (VIAPPL), a computer-mediated experimental platform designed to study social interaction and group processes^18^. We integrated agents into VIAPPL, enabling interactions between agents and human participants in a virtual environment. For clarity, we referred to both agents and humans who participated in the experiments as players, and only humans as participants. Both studies were approved by the Human Sciences Research Ethics Committee of the university.

## Study 1: nonadaptive agents

### Method

Agents in study 1, approved by the university ethics committee with a protocol reference number HSS/2210/018D, were preprogrammed to exhibit outgroup altruism by allocating resources to randomly selected outgroup members in each of 30 rounds of an exchange game. We refer to these agents as nonadaptive. All the experiments were performed in accordance with the university ethics committee guidelines and regulations.

#### Players (participants and agents), design and procedure

The players (N = 400) comprised 280 participants and 120 agents. The participants, university students, included 149 females and 131 males, aged eighteen years and above (M = 20, 41, SD = 2.83). The nonprobability, convenience sample was recruited using adverts and in person requests.

Participants signed an informed consent form, stipulating that they understood that participation was voluntary and confidential, and they were able to withdraw from the study at any point. We employed a multilevel design with rounds nested within individuals, and two four-person groups nested within games. We included five experimental conditions with 10 replicated games in each condition. The five conditions varied the number of agents in each group, as described in Table 2. Participants were randomly assigned to conditions and to groups within the condition but were told that the assignment was done based on the dot estimation task^39^ conducted prior to the experiment.

**Table 2:** The experimental conditions under which ingroup favoritism will be examined.

Condition	Short form	Description
Condition 1:0	1:0	One agent in one group, and no agent in the other group
Condition 2:0	2:0	Two agents in one group, and no agent in the other group
Condition 1:1	1:1	One agent per group
Condition 2:1	2:1	Two agents in one group, and 1 in the other group
Condition 2:2	2:2	Two agents per group

In the dot estimation task, each participant sat before a computer. Upon logging in, a message was displayed on the screen, explaining that they would be shown an image and asked to make a guess based on it. A circle containing a mix of blue and red dots appeared on the screen for 20 s. Participants were first asked to estimate the total number of dots within the circle and then to estimate the number of blue dots specifically. After submitting their estimates, a message informed them that they had been grouped with other players who made similar estimates.

**Figure 1:**
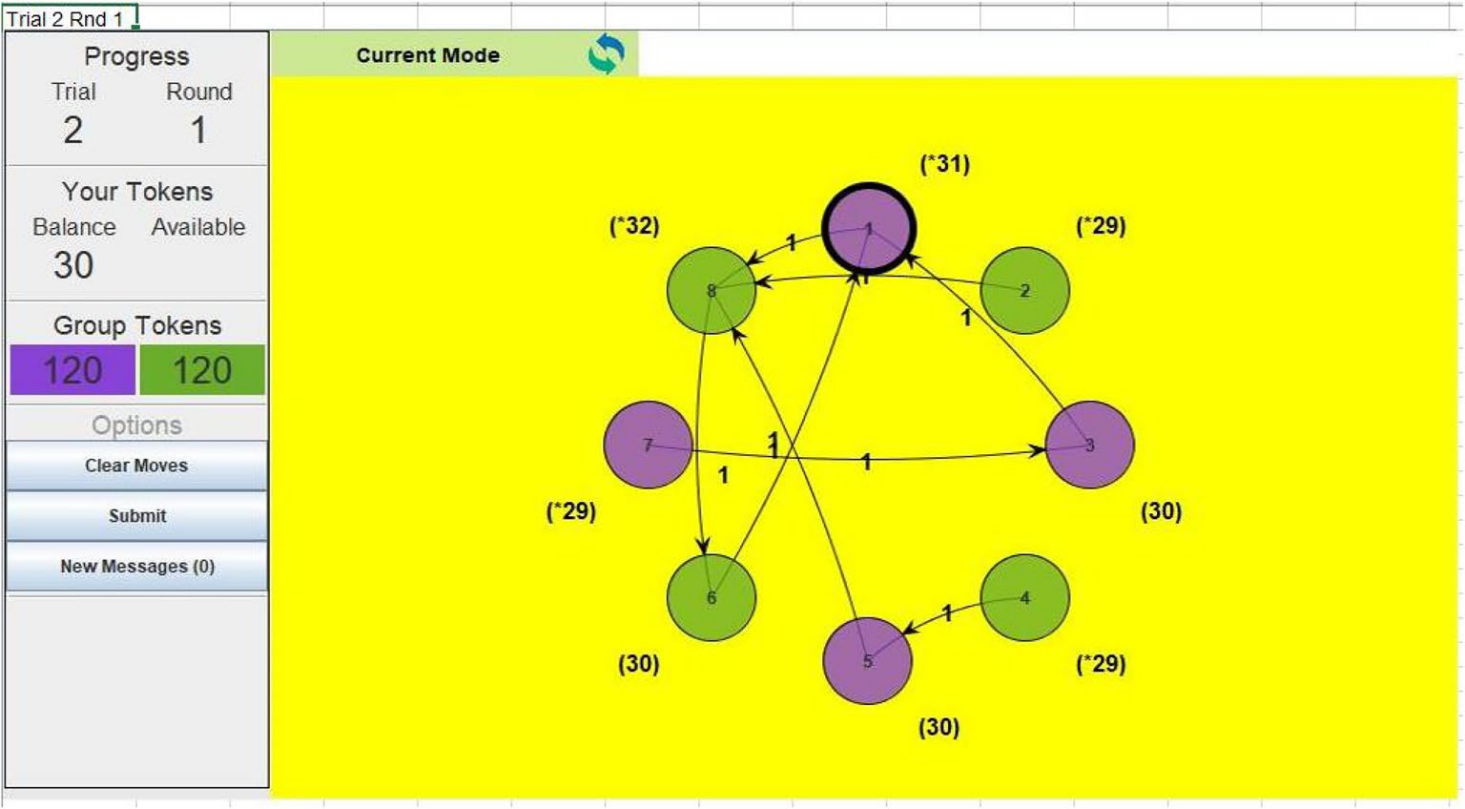
VIAPPL interface showing players’ token allocation. Each avatar (circles) represents a player. The thick outline indicates the player whose screen is shown.

The game replicated that reported by Durrheim, et al. ^18^, who showed that group identity promotes ingroup favoritism. Players received a starting allocation of 30 monetary tokens (total value R30/$1.60). Once the players had been assigned to a group, each computer displayed all players represented with avatars, specifically, circles arranged on their screens (See Fig. 1). We used the color of the circles to make the groups salient. Thus, participants were able to identify ingroup and outgroup players. However, they were unaware of the presence of artificial agents. The participants were instructed to allocate a token every round to any one of the eight players, 4 per group. For each game, a trial run (Trial 1) was conducted where each player played for two rounds before the actual game. This was done to ensure that players understood what was required of them. Each player's token balance is displayed on the screen. Each participant allocates a token by clicking the avatar representing the target recipient. Each token allocation increased the recipient’s token balance by one and decreased the allocator's token balance by one. At the end of each round, the exchanges and the token balance of each player were displayed on the screen and made visible to all participants (Fig. 1). To avoid the end-of-game effect, participants were not informed how many rounds the game would last. Similar to trivial giving, token allocation serves purely as an indicator of the willingness to cooperate.

We expected that agents’ outgroup altruism would reduce ingroup favoritism among both ingroup and outgroup members over time via reciprocity and imitation by human participants (H1: Ingroup favoritism). Due to the experimental nature of the study, minor deception was necessary as participants were led to believe, at the beginning of each game, that all the players were human. This low-risk deception was necessary to ensure that participants’ behavior did not change due to the awareness of the agents. Participants were debriefed at the end of the study, informing them of the presence of agents and that their group assignment was done randomly.

At the end of the game, participants rated the perceived fairness and humanness of other players. The fairness rating used a scale from 1 to 5, reflecting the participant’s judgment of whether the player being rated played fairly in the game. The higher the rating, the more convinced the participant was that the player played fairly. We applied the same scale to the humanness rating; a higher rating signifies more conviction that the player is a human rather than a computer program. The humanness rating was conducted very last to ensure that knowledge of agent players did not affect earlier behavior or the fairness rating.

We hypothesized that agents would be rated fairer than humans by outgroup players who were beneficiaries of their generosity but less fair by ingroup players because agents violated norms of ingroup favoritism (H2: Fairness). Additionally, we expected that there would be no difference between the ratings received by agents and those received by humans in the humanness rating. This manipulation check was conducted to ascertain whether participants were cognizant of the artificial agents.

#### Independent and dependent variables

The independent variables included Group (with two levels: group 1 and group 2), Condition (defined in Table 2), and Time (comprising five waves of 6 rounds each), while the dependent variables included ingroup favoritism, fairness rating, and humanness rating.

##### Ingroup favoritism

Equation 1 calculates ingroup favoritism (${IF}_{w}$) across each wave ($w$). We computed the ingroup to outgroup giving ratio for each player over the 6 rounds of each wave. The ratios were analyzed to observe trends over time and across experimental conditions. Ingroup, outgroup, and self-giving were count variables, ranging from 0 to 6 in each of the five waves, with self-giving considered a self-favoring strategy rather than a group-favoring one. Consequently, self-giving was not included in the ingroup favoritism calculation. Also, the data produced by agents were removed since the interest of the study was to investigate the effect of the agents’ behaviors on promoting outgroup altruism among humans.

1 $$IF_{w} = \frac{{\mathop \sum \nolimits_{i = tmin}^{tmax} IA_{i} }}{{\mathop \sum \nolimits_{i = tmin}^{tmax} (IA_{i} + OA_{i} )}}$$

Ingroup favoritism at wave w is in the range [0, 1], $t$ takes value from the range [1,30], specifying the actual round, while $tmix$ and $tmax$ take values from (1, 7, 13, 19, 25) and (6, 12, 18, 24, 30) respectively, thus specifying the minimum and maximum t for each wave. ${IF}_{w}$= ingroup favoritism at wave w, ${IA}_{i}$= ingroup allocation at round $i$, and ${OA}_{i}$= Outgroup allocation at round $i$. ${IA}_{i}$ is either 1 or 0. It is 0 when the token is allocated to an outgroup member and 1 when the token is allocated to an ingroup member. Also, ${OA}_{i}$ is 0 when the token is allocated to an ingroup member and 1 when the token is allocated to an outgroup member.

##### Fairness rating

The participants’ perceptions of fairness were assessed through a rating scale ranging from 1 to 5, indicating the extent to which they trusted that the rated player played fairly in the game. Higher ratings indicated a stronger belief that the player made fair allocations.

##### Humanness rating

We employed the same procedure for the humanness rating with one subtle change: higher ratings indicated a stronger belief that the player was a human (vs computer program).

#### Analysis

We employed multilevel modeling and utilized the Akaike Information Criterion (AIC) to assess the fitness of each model. Multilevel modelling is more suitable for the analysis due to hierarchical structure of the data^40^, and to account for random effects. For all the multilevel models, we conducted the analysis incrementally in the following steps. Firstly, we established statistical evidence for nesting, which included game level and individual level random effects. Secondly, we introduced a first-order autoregressive structure to account for repeated measures across waves. Lastly, we added fixed effect, two-way, and three-way interactions. The goodness of fit for each model developed at every step was recorded, and the model with the best fit was presented. See Appendix A of the supplementary materials for the presentation of the models’ comparisons. Additionally, descriptive statistics were provided to offer more information about some statistically significant tests. Confidence intervals (95%) were calculated using the bootstrap method, which is suitable when the data distribution is not normal or known. All analyses were performed in R, utilizing packages such as lmer and lme for a generalized linear mixed-effects model.

### Results

#### Ingroup favoritism

The best fit model (AIC = 90.34137, logLik of − 25.1707) presented in Table 3 incorporated a random effect at both the game and individual levels, a first-order autoregressive structure, fixed effects for the independent variables condition, group, and time, and two-way interactions.

**Table 3:** The Result of the multilevel analysis of ingroup favoritism in study 1.

	$\text{numDF}$	$\text{denDF}$	F-value	*P* value
Condition	4	45	1.2759	0.2936
Group	1	225	9.9406	0.0018
Time (wave)	1	1114	0.0104	0.9187
Condition: group	4	225	2.7623	0.0285
Condition: time	4	1114	1.9435	0.101
Group: time	1	1114	8.1999	0.0043

**Figure 2:**
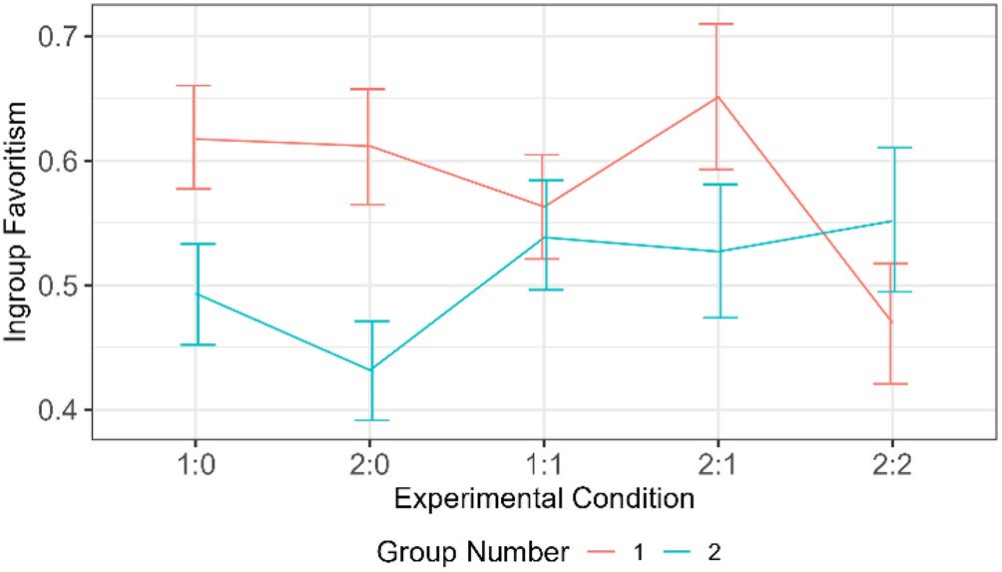
Differences in mean ingroup favoritism between conditions per group. The first digit (i.e., 1, 2, 1, 2, 2) in each experimental condition represents the number of agents in Group 1 while the second digit (i.e., 0, 0, 1, 1, 2) represents the number of agents in Group 2. There were four players per group.

**Figure 3:**
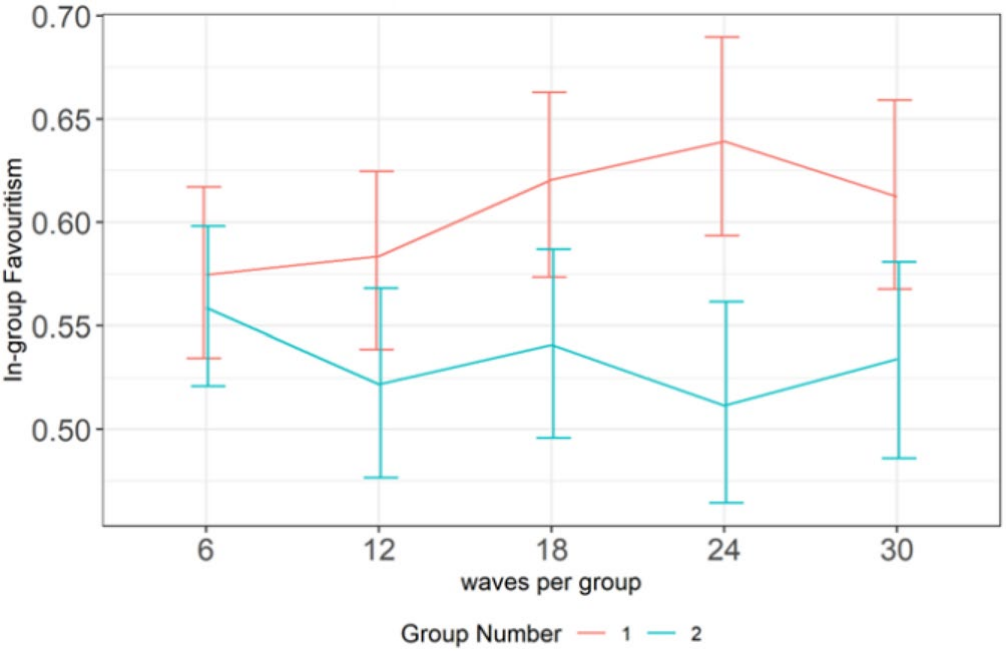
The difference in mean ingroup favoritism over time. Each wave comprised five rounds of exchanges.

Ingroup favoritism was statistically lower in groups with fewer agents (Group 2) than groups with more agents (Group 1) (Fig. 2). The difference in ingroup favoritism was notably high when Group 1 had two agents and Group 2 had no agent. This pattern indicates that agents successfully nudged outgroup altruism among outgroup members but not within the ingroup. Thus, invoking the feeling of reciprocity was a successful strategy but not imitation. Figure 3 shows that ingroup favoritism was significantly higher in Group 1 than in Group 2 at the fourth wave (x-axis mark 24).

#### Fairness and humanness rating

To assess the perceived fairness and humanness of agents and participants among ingroup and outgroup participants, we introduced two independent variables, RatingType and TargetPlayer. RatingType categorized ratings as InGroupRating (within the same group) or OutGroupRating (to a player from a different group), while TargetPlayer, distinguished ratings of humans and agents. The best fit fairness rating model (AIC = 6447.254, logLik =  − 3204.627) presented in Table 4 incorporated a random effect at both the game and individual levels, fixed effects for the independent variables, condition, RatingType, and TargtPlayer, and two-way interactions. An autoregressive structure was not included because there was no time factor in the fairness rating.

**Table 4:** The output of the fairness rating model.

	$\text{numDF}$	$\text{denDF}$	F-value	*P* value
(Intercept)	1	1657	4268.732	< 0.001
Condition	4	45	0.997	0.419
RatingType	1	1657	12.883	< 0.001
TargetPlayer	1	1657	3.597	0.058
Condition: RatingType	4	1657	1.49	0.203
condition:TargetPlayer	4	1657	4.892	< 0.001
TargetGroup:TargetParticipant	1	1657	11.535	< 0.001

**Figure 4:**
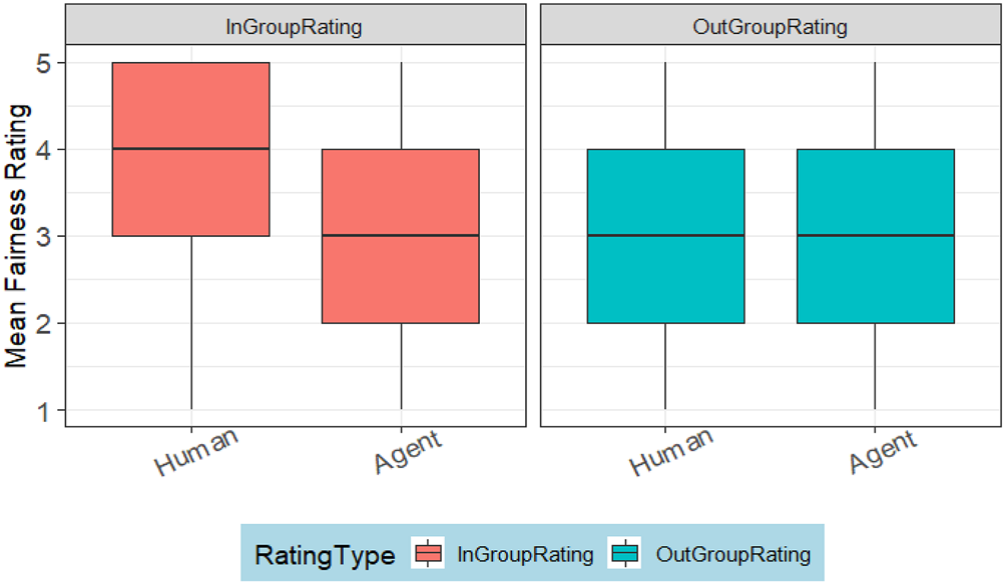
Fairness ratings of the ingroup members compared to the out-group members.

**Figure 5:**
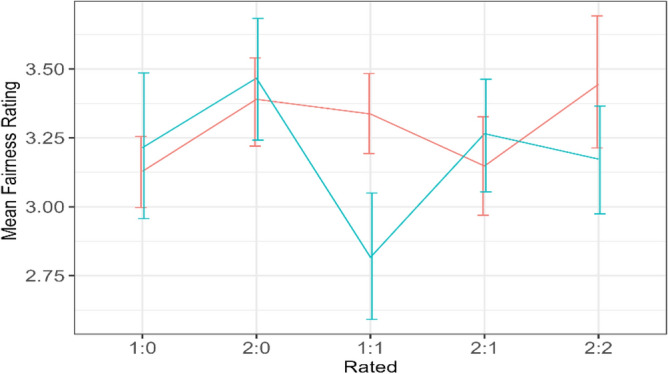
Mean fairness rating received by humans and agents. The green line shows the ratings received by agents while the red line shows that received by humans. The figure shows the interaction between rating type and condition.

The fairness ratings received by humans and agents did not differ significantly as indicated by the TargetPlayer variable. However, RatingType indicated a significant difference between ratings from out-group and ingroup members. Figure 4 illustrates that this difference occurs because participants rated ingroup participants fairer than others. Figure 5. plots the interaction effect of condition and target player. This shows that the significant effect was a result of humans being rated fairer in Condition 1:1.

Overall, the highest fairness ratings (M = 3.48, SD = 1.33) were received by humans from the ingroup members, followed by the ratings (M = 3.26, SD = 1.25) received by agents, from the outgroup members. The ratings (M = 3.11, SD = 1.31) received by humans from the outgroup members and that (M = 3.10, SD = 1.34) received by agents from the ingroup members have a negligible difference of 0.01, confirming that agents were treated as out-group members.

There was no significant difference (*p* = 0.447) between the ingroup and outgroup humanness ratings (Table 5). Also, there was no significant difference (*p* = 0.3964) between the humanness ratings received by humans and agents. The ratings (M = 2.76, SD = 1.49) and (M = 2.85, SD = 1.58) received by humans and agents respectively from the outgroup members are higher than those (M = 2.73, SD = 1.55) and (M = 2.75, SD = 1.55) received by humans and agents respectively from the ingroup members.

**Table 5:** The output of the humanness rating model.

	numDF	denDF	F-value	*P* value
(Intercept)	1	1646	4205.895	< .0001
Condition	4	45	0.435	0.7829
RatingType	1	1646	0.578	0.4472
TargetPlayer	1	1646	0.719	0.3965
condition:RatingType	4	1646	0.439	0.7804
condition:TargetPlayer	4	1646	7.879	< .0001
RatingType:TargetPlayer	1	1646	0.181	0.6705
condition:RatingType:TargetPlayer	4	1646	2.578	0.0359

#### Test for withdrawing cooperation

To examine whether ingroup members refrained from cooperating with the ingroup agents (defectors), we examined the differences in allocations from participants to ingroup humans, outgroup humans, ingroup agents and outgroup agents. Due to differences in the number of agents in each group, we normalized the allocations for each category $x$ as ${n}_{x}/{N}_{x}$, where ${n}_{x}$ represents the number of allocations to x, and ${N}_{x}$ represents the total number of possible allocations to x. For example, a group with 3 humans and 1 agent will have ${N}_{x}$ = 3 for ingroup agent, outgroup human, and outgroup agent, and ${N}_{x}$ = 2 for ingroup human because self-allocations were removed.

**Figure 6:**
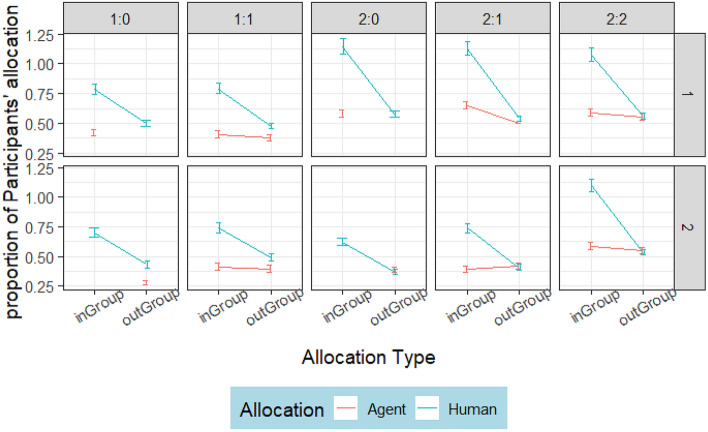
Allocation from participants to ingroup and outgroup humans and agents showing high cooperation with ingroup humans and low cooperation with ingroup agents.

A multilevel model (AIC =  − 333.2289, df = 38, logLik = 204.6144) shows a significant (*p* < 0.001) difference in the participants’ allocation to agents and humans across conditions. Table 6 shows significant differences in the participants’ cooperation with agents and other humans within the ingroup and the outgroups. Note that there was no significant interaction (*p* = 0.235) between condition and target player, or (*p* = 0.244) between group and target player. Participants cooperated more with ingroup humans than with ingroup agents across all conditions and groups (see Fig. 6). The nonparallel lines in Fig. 6 show a significant interaction, with participants showing ingroup favoritism toward ingroup human participants, but not ingroup agents or outgroup players. The difference between the average (M = 0.818, SD = 0.358) allocations to ingroup humans and (M = 0.522, SD = 0.207) ingroup agents is 0.296.

**Table 6:** The output of the cooperation model.

	$\text{numDF}$	$\text{denDF}$	F-value	*P* value
Condition	4	72.5	77.2102	< 0.0001
Group	1	5590.2	317.0188	< 0.0001
targetPlayer	1	5583.6	911.5452	< 0.0001
allocationType	1	5602.7	867.8244	< 0.0001
Condition:Group	4	5587.1	86.3577	< 0.0001
Condition:targetPlayer	4	5593.3	1.3903	0.2345
Condition:allocationType	4	5592.2	27.2054	< 0.0001
Group:targetPlayer	1	5602.6	1.3556	0.2443
Group:allocationType	1	5605.3	49.7584	< 0.0001
targetPlayer:allocationType	1	5597.9	527.5546	< 0.0001
Condition:Group:targetPlayer	4	5600	16.3155	< 0.0001
Condition:Group:allocationType	4	5595.8	20.9376	< 0.0001
Condition:targetPlayer:allocationType	2	5598.6	17.8482	< 0.0001
Group:targetPlayer:allocationType	1	5600.7	0.3759	0.5398

### Discussion

The result of Study 1 shows that outgroup altruism nudged by nonadaptive agents increased intergroup cooperation among the outgroup humans, but not among the ingroup. This result indicates that invoking the feeling of reciprocity was effective. Ingroup favoritism was higher in groups with more agents than groups with fewer agents. For example, in Fig. 2, Condition 1:0 (i.e., one agent in Group 1 and none in Group 2), ingroup favoritism was higher among Group 1 members than it was among Group 2 members. This high ingroup favoritism among groups with more agents suggests that participants did not imitate the agents. Thus, social comparison was not an effective nudge mechanism in Study 1. The result of the fairness rating, which shows that ingroup humans were rated higher than ingroup agents, indicates that participants find ingroup members who favor outgroup members unfair, i.e., outgroup favoring ingroup members earn a bad reputation. This interpretation follows from Duradoni, et al. ^38^, who suggest that fairness is an important component on which people base their social behavior. Although the result of the humanness ratings shows that agents were not identified, ingroup members reinforced ingroup favoritism and withheld cooperation with the ingroup agents.

Based on the result of Study 1, we conclude that participants did not imitate the agents because the agents were perceived as violating the ingroup favoritism norm. Participants perceived ingroup agents as norm violators due to the agents’ outgroup altruistic behavior. The preprogrammed behavior did not allow agents to associate with ingroup members and maintain a good reputation within the ingroup. We hypothesized that incorporating a strategy that could adaptively promote ingroup identity while nudging outgroup altruistic norms would reduce ingroup favoritism in both ingroup and outgroup members and improve cooperation with ingroup agents.

## Study 2: adaptive agents

Drawing from the limitations of Study 1, Study 2 employed adaptive agents whose altruistic behaviors were informed by the prediction of a machine learning algorithm, specifically, the Cluster Hidden Markov Model (CHMM) described in Igwe and Durrheim^41^. In the C-HMM, each state is a cluster representing one of the identified possible motives for token allocation. These motives were derived from the observed features of participants’ previous allocations within the experimental game. These features include participants’ group identities, ingroup and outgroup ties that indicate the participants’ relationships with their ingroup and outgroup, starting tokens that indicate participants’ wealth, and participants’ tendency to reciprocate^41^. Thus, each state within the CHMM corresponds to a motive, with transitions between states representing shifts in social motives based on observed behaviors and outcomes. An observation at each state represents a prediction of whether a player will reciprocate in a given round as the game progresses. Specifically, the output of the CHMM consists of the predicted probability distributions for transitions between motives and the likelihood of reciprocating allocations when possessing the motive. Predictions were not necessary in the first round of each game, as the model would not have data from which to learn^41^. We refer interested readers to^41^ for a detailed description of CHMM.

The use of machine learning prediction allowed the adaptive agents to interact with the ingroup when it predicted that interaction with the outgroup would not promote outgroup altruism. We present details of how adaptive agents use the CHMM in the design and procedure section.

### Method

We employed the same methodology used in Study 1. We explicitly outlined modifications made along with the rationale behind each modification.

#### Participants and agents

Players (N = 480) in Study 2 comprised 120 agents and 360 university students as participants, which included 261 Female and 99 Male. Participants were 18 years and above (M = 22. 24, SD = 3.20). We employed the same recruitment and sampling methods used in Study 1.

#### Design and procedure

We employed the same design as Study 1, with two modifications. First, the descriptive analysis of the interaction effects between condition and group in Study 1 (Fig. 2) reveals a more pronounced (and significant) disparity in ingroup favoritism when the groups contain an imbalanced number of agents. Conversely, this disparity is reduced (and statistically non-significant) when the number of agents in the groups is balanced. Based on this knowledge, Study 2 examined ingroup favoritism in a simplified three condition design: a null condition—no agents, Condition 2:0—two agents in Group 1 and none in Group 2, and Condition 2:2—two agents in each group. These conditions represent control, the opportunity for more disparity, and less disparity in ingroup favoritism, respectively. Second, although there was no significant increase in ingroup favoritism over time in Study 1, prior research^42^ has indicated that merely ten rounds are sufficient for the emergence of a norm. Thus, we concluded that the assertion by Titlestad, et al.^42^ holds in our case; otherwise, there would have been a significant change in ingroup favoritism over the 30 rounds played in Study 1. Consequently, we reduced the number of rounds from 30 to 15. The experiments comprised 60 games, with 20 replications per condition, each consisting of three waves of five rounds of token allocation. Participants were randomly assigned to groups using the same procedure as in Study 1. They were given 40 monetary tokens at the start of the game and were required to allocate one to any player in the game each round.

In each round of a game, each adaptive agent used the CHMM^41^ to identify outgroup participants likely to reciprocate. Then, the agent allocated its token randomly to one of these identified participants. Where an agent found no outgroup participant that was likely to reciprocate, the agent allocated its token randomly to an ingroup participant. This way, Study 2 attempted to promote cross-group reciprocity while trying to change the agent’s ingroup members’ perception of ingroup cooperation.

#### Informed consent

Participants signed an informed consent form prior to participating in the study.

### Results

The analysis of the adaptive agent’s allocation (Table 7) shows that agents allocated more tokens to the outgroup than the ingroup members.

**Table 7:** Agents allocation to ingroup and outgroup members for each condition and group that includes agents.

Condition	Agents group	Ingroup (ration)	Outgroup (ratio)
2:0	1	176 (29.33%)	424 (70.67%)
2:2	1	162 (27.00%)	438 (73.00%)
2:2	2	195 (32.50%)	405 (66.50%)

#### Ingroup favoritism

A multilevel model (AIC = 519.8335, logLik =  − 245.9168) built with two-way interactions and random effect at both the individual and group level shows that experimental condition had significant effect on ingroup favoritism. Group was not significant as shown in Table 8. However, the interaction between group and condition was statistically significant (*p* = 0.011). We explored these effects via descriptive analysis.

**Table 8:** The Result of the multilevel analysis of ingroup favoritism in study 2.

	numDF	denDF	F-value	*p* value
(Intercept)	1	716	762.3867	< .0001
Condition	2	57	5.4466	0.0068
Group	1	297	1.4753	0.2255
Time	1	716	17.2718	< .0001
Condition:Group	2	297	4.5652	0.0111
Condition:Time	2	716	5.5952	0.0039
Group:Time	1	716	0.5256	0.4687

**Figure 7:**
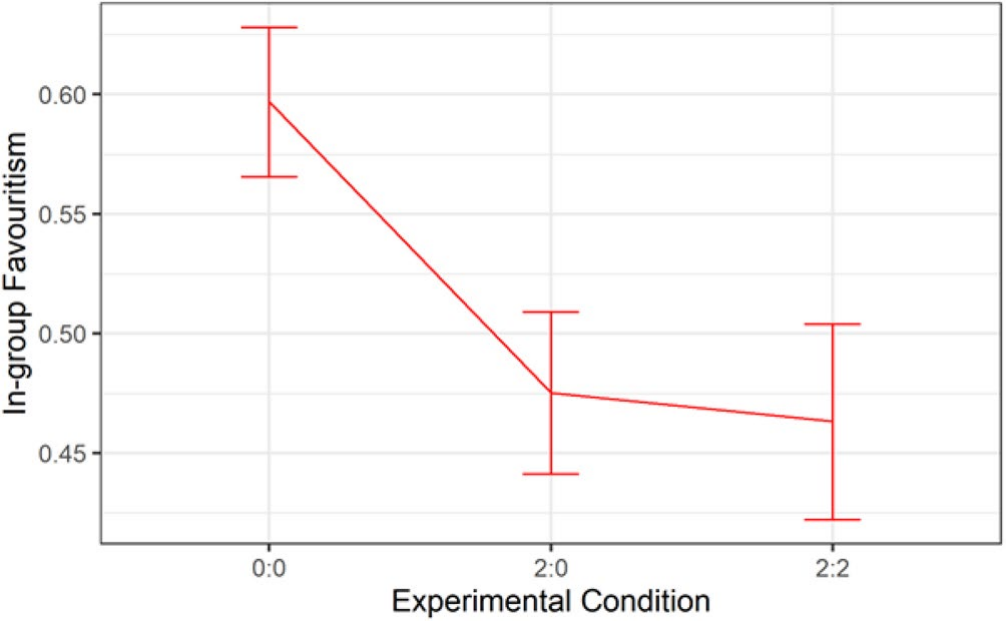
Differences in mean ingroup favoritism between conditions.

As we expected, the null condition has high ingroup favoritism as shown in Fig. 7. However, the high ingroup favoritism was significantly reduced in conditions 2:0 and 2:2. There was no significant difference in ingroup favoritism in conditions 2:0 and 2:2. Thus, it is evident that agents in Study 2 were able to weaken ingroup favoritism, compared to agents in Study 1.

**Figure 8:**
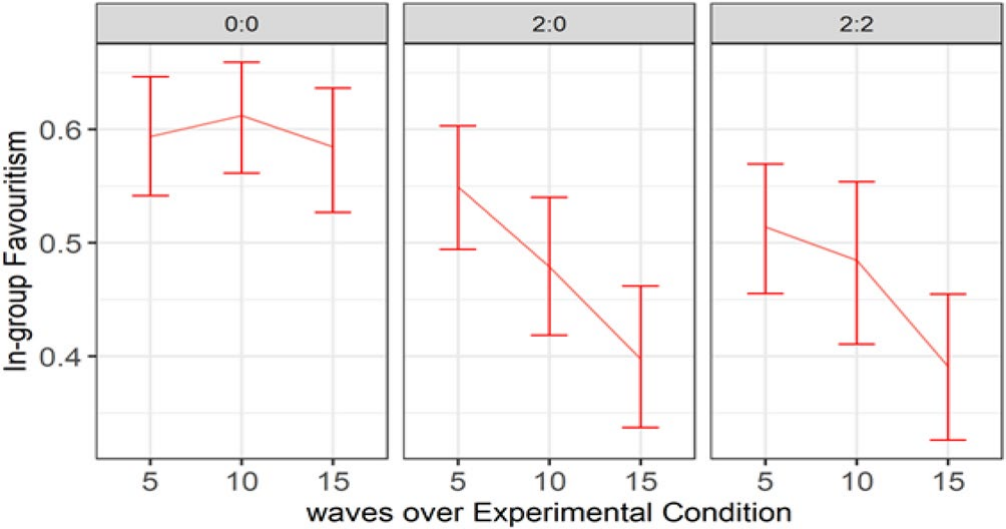
A high Ingroup favoritism in the null condition which persisted overtime vs ingroup favoritism in conditions 2:0 and 2:2 which reduced over time.

**Figure 9:**
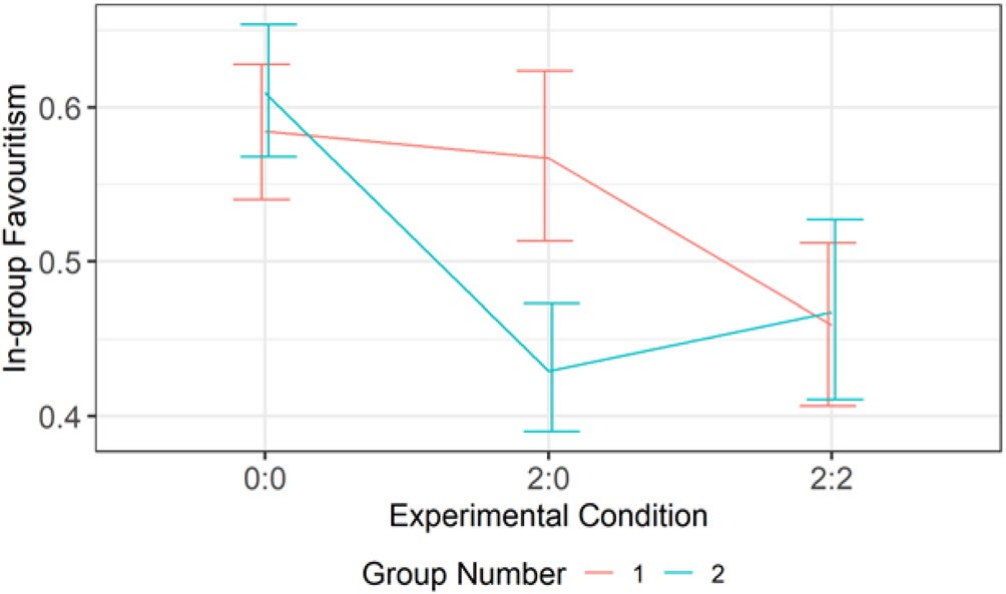
Differences in mean ingroup favoritism between conditions per group.

Figure 8 plots the graph of the interaction between Condition and Round (Time). Ingroup favoritism was higher in the null condition and relatively stable over time. It decreased over time in conditions 2:0 and 2:2, with main effect between wave 1 and wave 3. The trend implies that the agents’ ability to reduce ingroup favoritism increased over time. This implication was confirmed by a significant difference (*p* = 0.003) between waves 1 and 3 in Condition 2:0, and that (*p* = 0.027) between waves 1 and 3 in Condition 2:2. Figure 9 shows that the reduced ingroup favoritism found in Condition 2:0 was mainly from Group 2, the group with no agent, who benefited from the outgroup altruism of the agents in Group 1.

#### Fairness and humanness rating

**Figure 10:**
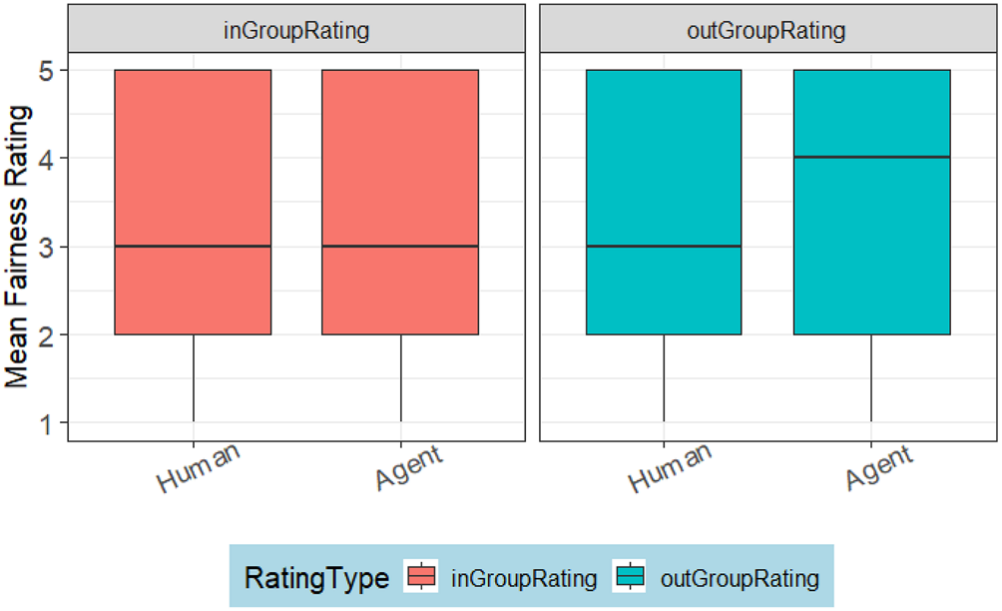
Outgroup agents were rated fairer than humans.

We processed the fairness ratings as described in Study 1. A best fit model (AIC = 4711.299, logLik =  − 2348.650) presented in Table 9 was built with a fixed effect of Condition (*p* = 0.343), RatingType (*p* = 0.553) and TargtPlayer (*p* = 0.020). The model shows a significant difference between the ratings received by humans and agents. A descriptive analysis (Fig. 10) of this significant difference shows that outgroup agents were rated fairer than humans.

**Table 9:** The output of the fairness rating model in study 2.

	numDF	denDF	F-value	*p* value
(Intercept)	1	1185	1140.3792	< .0001
Condition	1	38	0.9239	0.3425
TargetGroup	1	1185	0.3523	0.5529
TargetParticipant	1	1185	5.4441	0.0198

**Figure 11:**
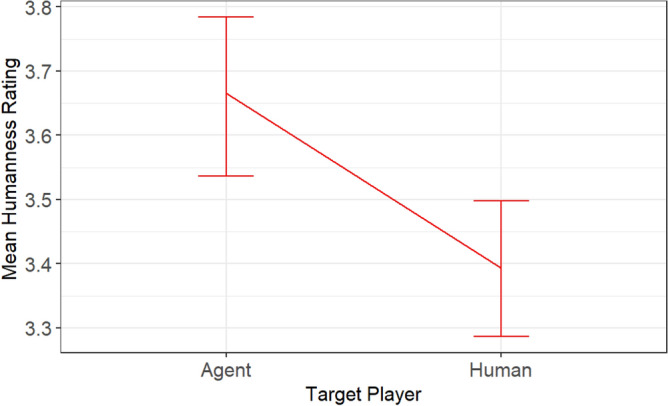
The differences in mean humanness ratings received by humans and agents.

Furthermore, the humanness rating model (AIC = 5143.925, logLik =  − 2564.963) presented in Table 10 indicates that neither Condition (*p* = 0.635) nor RatingType (*p* = 0.517) achieved statistical significance. Nevertheless, we observed a significant difference in the ratings between agents and humans (*p* = 0.001). Descriptive analysis (Fig. 11) of the difference revealed that agents were rated as more human than participants. Considering that a rating of 5 indicates the participant perceives the player as human, while a rating of 1 suggests the participant believes the player is an artificial agent, this result implies that participants were more confident that agents were more human than the participants.

**Table 10:** The output of the humanness rating model in study 2.

	numDF	denDF	F-value	*p* value
(Intercept)	1	1188	3254.949	< .0001
Condition	1	38	0.229	0.6347
TargetGroup	1	1188	0.420	0.5171
TargetParticipant	1	1188	10.545	0.0012

#### Test for withholding cooperation

While the fairness and humanness ratings reflected a positive impression of the adaptive agents, we explored whether the participants’ impression transpired into less or more cooperation with the adaptive agents. The best fit multilevel model (AIC =  − 874.8916, logLik = 453.4458) presented in Table 11 indicates that the difference between the allocations to agents and humans is not significant (*p* = 0.177). However, the interaction effect of group, allocation type, and target player was significant (*p* = 0.006) .

**Table 11:** The output of cooperation model in study 2.

	numDF	denDF	F-value	*p* value
Condition	1	2371.6	29.6992	< .0001
Group	1	3987.8	32.3504	< .0001
targetPlayer	1	3964.2	1.8176	0.1776
allocationType	1	3964.2	2.8156	0.0934
Condition:Group	1	3984.9	32.5277	< .0001
Condition:targetPlayer	1	3964.2	0.2587	0.6110
Condition:allocationType	1	3964.2	2.4453	0.1179
Group:targetPlayer	1	3964.2	8.5	0.0035
Group:allocationType	1	3964.2	6.7916	0.0091
targetPlayer:allocationType	1	3964.2	0.7221	0.3954
Condition:Group:targetPlayer	1	3964.2	0.5915	0.4418
Condition:Group:allocationType	1	3964.2	4.0281	0.0448
Group:targetPlayer:allocationType	1	3964.2	7.5067	0.0061

**Figure 12:**
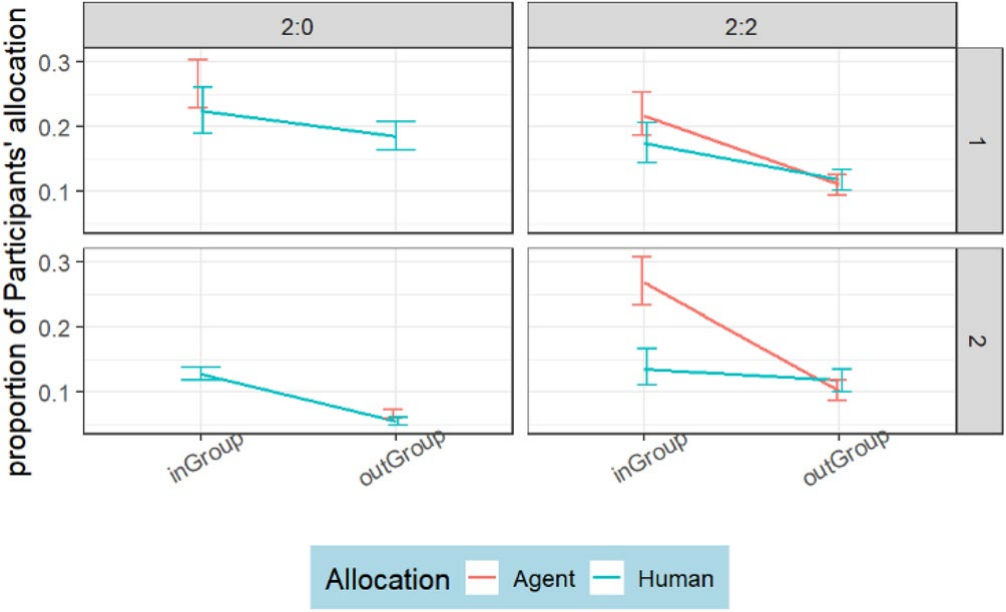
Allocation from humans to ingroup and outgroup humans and agents. The headings, indicated as 2:0 and 2:2, represent conditions 2:0 and 2:2 respectively. The graph shows no significant difference except in one group of condition 2:2.

Descriptive analysis in Fig. 12 shows no difference in allocation to ingroup humans and ingroup agents in Condition 2:0. Interestingly, ingroup participants allocated more tokens to ingroup agents than ingroup humans in Group 2 of Condition 2:2. This result obtained from Group 2 of Condition 2:2 shows a slight improvement over Study 1, where ingroup participants allocated more tokens to the outgroup agents than the ingroup agents. The average normalized allocation to ingroup humans (M = 0.165, SD = 0.245) and ingroup agents (M = 0.249, SD = 0.309) differs by 0.084. This is 20% more ingroup allocation to adaptive agents than humans, showing improved cooperation with agents. Notably, there was no significant difference between normalized allocation to outgroup agents and outgroup humans.

### Discussion

Study 2 aimed to investigate adaptive agents’ ability to reduce ingroup favoritism, improve participants’ cooperation with ingroup agents, and intergroup cooperation during intergroup interaction. Overall, the agents in Study 2 weaken ingroup favoritism more strongly amongst outgroup than ingroup participants. Evidence from Fig. 9 shows that the reduced ingroup favoritism resulted from invoking the feeling of reciprocity, not social comparison; otherwise, ingroup humans would have imitated ingroup agents in condition 2:0. However, ingroup participants allocated more tokens to ingroup agents than ingroup humans in condition 2:2 of Fig. 12. This result reflects the importance of maintaining a good relationship with the ingroup while promoting outgroup altruism. Although, the relationship did not lead ingroup participants to imitate agents’ outgroup altruism, it was reflected in the fairness rating. Agents were rated fairer than humans (Fig. 10) and more human than the human participants (Fig. 11).

## General discussion

We aimed to increase outgroup altruism by introducing non-human agents into an intergroup exchange environment, and elucidate how agents achieve the goal by examining the reciprocity and imitation mechanisms. We sought to promote outgroup cooperation by two nudge mechanisms: invoking the feeling of reciprocity and social comparison. Outgroup altruism was nudged via nonadaptive agents (Study 1), and adaptive (Study 2). Study 2 aimed to evaluate whether a good relationship with ingroup members would enhance the likelihood of ingroup members imitating outgroup altruistic behavior. Informed by a machine learning algorithm, adaptive agents dynamically maintained a good relationship with both ingroup and outgroup members.

In the first hypothesis (H1), we expected that agents’ outgroup altruism would reduce ingroup favoritism via imitation and invoking the feeling of reciprocity. Although both Study 1 and Study 2 demonstrate that invoking the feeling of reciprocity rather than imitation reduced ingroup favoritism, adaptive agents in Study 2 had a greater impact on reducing ingroup favoritism. Thus, H1 was partly supported by both studies. In Study 1, participants did not imitate ingroup agents; instead, they withdrew cooperation from ingroup agents. This contrasts with our observation in Study 2, where participants neither imitated the agents nor withdrew cooperation from ingroup agents. by ingroup members but benefited from ingroup favoritism.

In the second hypothesis (H2), we expected that participants would rate outgroup agents as fairer than humans, and ingroup agents as less fair than ingroup humans. Indeed, Study 1 supports the latter; however, there was no evidence supporting the former. This suggests that humans did not acknowledge the outgroup generosity of ingroup agents. The result underscores that, in Study 1, ingroup members reinforced ingroup favoritism. In contrast to Study 1, participants in Study 2 rated outgroup agents as fairer than humans, as expected. Interestingly, there was no evidence that participants rated ingroup humans as fairer than ingroup agents, highlighting the importance of maintaining a good reputation within the ingroup while promoting outgroup altruism.

In our manipulation check, we expected that there would be no difference between the ratings received by agents and those received by humans. In other words, participants were not expected to identify the agents in the experiment. Study 1 supports this expectation. Thus, in Study 1, participants’ allocation decisions were not influenced by the presence of agents. However, we were intrigued by the fact that, in Study 2, participants rated agents as more human than humans.

While promoting outgroup altruism via social comparison undermined the reputation of ingroup agents in Study 1, leading participants to withdraw cooperation from these agents, participants in Study 2 did not withhold cooperation from ingroup agents, as these agents maintained a good reputation within the ingroup. We conclude that the proportion of tokens agents allocated to the ingroup in Study 2, was not sufficient to prompt imitation. However, it was adequate to maintain a good reputation within the ingroup and create a sense of fairness among the participants.

## Implications and limitations

Both these studies have several implications for research on group identity, reputation, group dynamics and interdisciplinary research. Group Identity: Study 1 suggests that violating the ingroup favoritism norm can create undesirable outcomes amongst ingroup members, leading to withholding of cooperation. Thus, obeying ingroup norms can be perceived as upholding one’s group identity. Adaptive agents identified with the ingroup by allocating some tokens to ingroup members (see Table 7). Thus, the agents upheld their group identity while promoting outgroup altruism, facilitating participants’ cooperation with the agent. Reputation: Agents in Study 1 did not maintain a good reputation within the ingroup, resulting in humans rating ingroup humans fairer than ingroup agents. Study 2 reinforces research on fairness and its implications on reputation. It provides evidence that supports Lee, et al.^43^ assertion that the perception of fairness within an exchange context reinforces trust, which leads to a good reputation. Group dynamics: Study 2 demonstrates the use of machine learning-based agents as experimental stooges in nudging outgroup altruism while upholding participants’ cooperation with agents. It shows that machine learning agents, which make decisions based on the prediction of participants’ behavior in the game, were able to interact adaptively with both the ingroup and the outgroup members. Interdisciplinary Research: Study 2 shows that the perception of fairness can enhance the perception of humanity in an artificial agent (see Appendix B of the supplementary material for the regression analysis). This can enhance research in other fields such as healthcare, where robots interact with patients, especially with the recent escalating use of AI in almost every sphere of life.

While these experiments have real-world implications, they may not generalize to real world contexts involving a higher degree of complexity. The participants’ behaviors may not be representative of the broader population but a particular social group. Furthermore, Study 2 used a machine learning model. The internal processes of the algorithms may not be fully explainable. Thus, there may be variables that influence the prediction but are not explained or accounted for due to the nature of the algorithm. Research could be developed towards improving the algorithm that controls the agents’ decision making.

### Supplementary Information

Supplementary Information.

## Data Availability

The data analyzed in this study can be accessed via the linked online public repository(https://osf.io/vxhm6/?view_only=c46bbf9260e140a6a21e3f11a6ee6be4).
