# Supplementary material for: Using artificial agents to nudge outgroup altruism and reduce ingroup favoritism in human-agent interaction
Source: Sci Rep. 2024 Jul 9;14:15850. doi: 10.1038/s41598-024-64682-5 (PMC11233637; doi:10.1038/s41598-024-64682-5)
Supplement: Supplementary file 1 — Supplementary Information. [file 41598_2024_64682_MOESM1_ESM.pdf]

Using Artificial Agents to Nudge Outgroup Altruism and Reduce Ingroup Favoritism in Human-Agent Interaction

Kevin Igwe[1,*], Kevin Durrheim[1,+]

[1]Department of Psychology, Faculty of Humanities, University of Johannesburg, Bunting Road, Auckland Park 2092, Johannesburg, South Africa
*igwekevin@gmail.com;
+kevind@uj.ac.za

Appendix A: Model comparison

Tables S1 through S8 present the model comparison statistics that led to the chosen models in the studies. The AIC (Akaike's Information Criteria), BIC (Bayesian Information Criteria), and LogLik (log-likelihood ratio) are model fit statistics that were used for comparison. AIC balances the goodness of fit of the model with the complexity of the model (measured by the number of parameters). The lower value of AIC is preferred. BIC is similar to AIC but with a stricter penalty for model complexity. Also, a lower value of BIC is preferred. Lastly, LogLik measures how well the model fits the data. A higher value implies a better model.

We used the factor() function in R to convert categorical variables into factors. This function assigns integer codes to each level of the categorical variable, starting from 1 for the first level, 2 for the second, and so on. For example, conditions 0:0, 2:0, and 2:2 were assigned 1, 2, and 3, respectively. We consider a model with a better score in at least two of the metrics as a better model. In the Test column, a better model is highlighted in bold font. The "Model Type" explains the composition of the model, i.e., the random effect, interclass correlation, main effects, and interactions where possible.

Table S1. Ingroup favoritism models comparison statistics (Study 1)

Model #	Model Type	AIC	BIC	LogLik	Test	P-value
1	No random effect	529.798	540.2865	-262.899		
2	Random effect at the game level	477.9833	493.716	-235.9917	1 vs **2**	< .0001
3	2 + Random effect at the individual level	116.6706	137.6476	-54.33532	2 vs **3**	< .0001
4	3 + ICC at the game and individual levels	101.2406	127.4618	-45.62031	3 vs **4**	< .0001
5	4 + condition, group, and time as main effects	98.97578	156.6623	-38.48789	4 vs **5**	0.0268
6	4 + two-way interactions	**90.34137**	**195.2259**	-25.17068	5 vs **6**	0.0016
7	4 + three-way interactions	96.51265	222.3741	-24.25633	**6** vs 7	0.7672

Table S2. Fairness rating models comparison statistics (Study 1)

Model #	Model Type	AIC	BIC	LogLik	Test	P-value
1	No random effect	6606.327	6617.475	-3301.164		
2	Random effect at the game and individual levels	6562.839	6579.560	-3278.420	1 vs **2**	< .0001
3	2 + Random effect at the individual level	6474.303	6496.597	-3233.151	2 vs **3**	< .0001
4	3 + Condition, RatingType and TargetPlayer as main effects	6466.252	6521.987	-3223.126	3 vs **4**	0.0027
5	3 + two-way interactions	**6447.254**	**6553.151**	-3204.627	4 vs **5**	< .0001
6	3 + three-way interactions	6451.412	6579.604	-3202.706	**5** vs 6	0.4278

Table S3. Humanness rating models comparison statistics (Study 1)

Model #	Model Type	AIC	BIC	LogLik	Test	P-value
1	No random effect	7151.047	7162.187	-3573.524		
2	Random effect at the game and individual levels	7134.300	7151.010	-3564.150	1 vs **2**	< .0001
3	2 + Random effect at the individual level	7119.979	7142.258	-3555.989	2 vs **3**	0.0001
4	3 + Condition, RatingType and TargetPlayer as main effects	7128.970	7184.670	-3554.485	**3** vs 4	0.8078
5	3 + two-way interactions	7113.697	7219.525	-3537.848	3 vs **5**	0.0001
6	3 + three-way interactions	**7111.307**	7239.415	-3532.653	5 vs **6**	0.0344

Table S4. Model comparisons for humans withholding cooperation (Study 1). We modeled the normalized allocations received by the players as the dependent variable, while the independent variables are **Condition**, **Group** (the players' assigned group, i.e., Group 1 or Group 2), **allocationType** (received by ingroup or outgroup member), **TargetPlayer** (received by Human or Agent).

Model #	Model Type	AIC	BIC	LogLik	Test	P-value
1	No random effect	2566.85	2580.11	-1281.42		
2	Random effect at the game level	2505.83	2525.73	-1249.91	1 vs **2**	< .0001
3	2 + Condition, Group, allocationType, and TargetPlayer as main effects	534.52	600.85	-257.26	2 vs **3**	< .0001
4	2 + two-way interactions	-205.40	-159.58	127.70	3 vs **4**	< .0001
5	2 + three-way interactions	**-333.23**	-142.83	**204.61**	4 vs **5**	< .0001
6	2 + four-way interactions	-322.20	-297.95	313.00	**5** vs 6	0.3033

Table S5 Ingroup favoritism models comparison statistics (Study 2)

Model #	Model Type	AIC	BIC	LogLik	Test	P-value
1	No random effect	728.0316	738.0010	-362.0158		
2	Random effect at the game level	617.5354	632.4896	-305.7677	1 vs **2**	< .0001
3	2 + Random effect at the individual level	550.2124	570.1513	-271.1062	2 vs **3**	< .0001
4	3 + ICC at the game and individual levels	550.5159	575.4395	-270.2580	**3** vs 4	0.1927
5	4 + condition, group, and time as main effects	530.4957	575.3582	-256.2479	3 vs **5**	< .0001
6	4 + two-way interactions	**519.8335**	589.6196	**-245.9168**	5 vs **6**	0.0009
7	4+ three-way interactions	521.5605	601.3160	-244.7803	**6** vs 7	0.3209

Table S6. Fairness rating models comparison statistics (Study 2)

Model #	Model Type	AIC	BIC	LogLik	Test	P-value
1	No random effect	5004.060	5014.530	-2500.030		
2	Random effect at the game and individual levels	4861.364	4877.069	-2427.682	1 vs **2**	< .0001
3	2 + Random effect at the individual level	4712.015	4732.954	-2352.007	2 vs **3**	< .0001
4	3 + Condition, RatingType and TargetPlayer as main effects	**4711.299**	4747.944	**-2348.650**	3 vs **4**	0.0815
5	3+ two-way interactions	4714.448	4766.797	-2347.224	**4** vs 5	0.4151
6	3 + three-way interactions	4715.743	4773.327	-2346.872	**4** vs 6	0.4694

Table S7. Humanness rating models comparison statistics (Study 2)

Model #	Model Type	AIC	BIC	LogLik	Test	P-value
1	No random effect	5165.340	5175.814	-2580.670		
2	Random effect at the game and individual levels	5150.171	5165.883	-2572.086	1 vs **2**	< .0001
3	2 + Random effect at the individual level	5149.098	5170.046	-2570.549	2 vs **3**	0.0796
4	3 + Condition, RatingType and TargetPlayer as main effects	**5143.925**	5180.585	**-2564.963**	3 vs **4**	0.0108
5	3 + two-way interactions	5145.092	5197.462	-2562.546	**4** vs 5	0.1844
6	3 + three-way interactions	5146.321	5203.929	-2562.161	**4** vs 6	0.2308

Table S8. Table S4. Model comparisons for humans withholding cooperation (Study 2). We modeled the normalized allocations received by the players as the dependent variable, while the independent variables are **Condition**, **Group** (the players' assigned group, i.e., Group 1 or Group 2), **allocationType** (received by ingroup or outgroup member), **TargetPlayer** (received by Human or Agent).

Model #	Model Type	AIC	BIC	LogLik	Test	P-value
1	No random effect	-625.3803	-612.7848	314.6902		
2	Random effect at the game level	-623.78	-604.89	314.89	1 vs **2**	0.5264
3	2 + Condition, Group, allocationType, and TargetPlayer as main effects	-825.20	-781.12	419.60	2 vs **3**	< .0001
4	2 + two-way interactions	-867.07	-805.19	446.53	3 vs **4**	< .0001
5	2 + three-way interactions	**-874.89**	-774.12	**453.45**	4 vs **5**	0.0350
6	2 + four-way interactions	-874.89	-774.12	453.45	**5** vs 6	0

Appendix B: Regression analysis for fairness and humanness rating in Study 2.

Intrigued by the remarkable outcome of Study 2, we conducted a regression analysis to investigate the correlation between humanness and fairness ratings in both humans and agents. In both conditions (2:0, and 2:2) involving agents, Fig. S1 shows that the perception of fairness can enhance the perception of humanity in an artificial agent. Thus, humanness is predicted by fairness in the artificial.

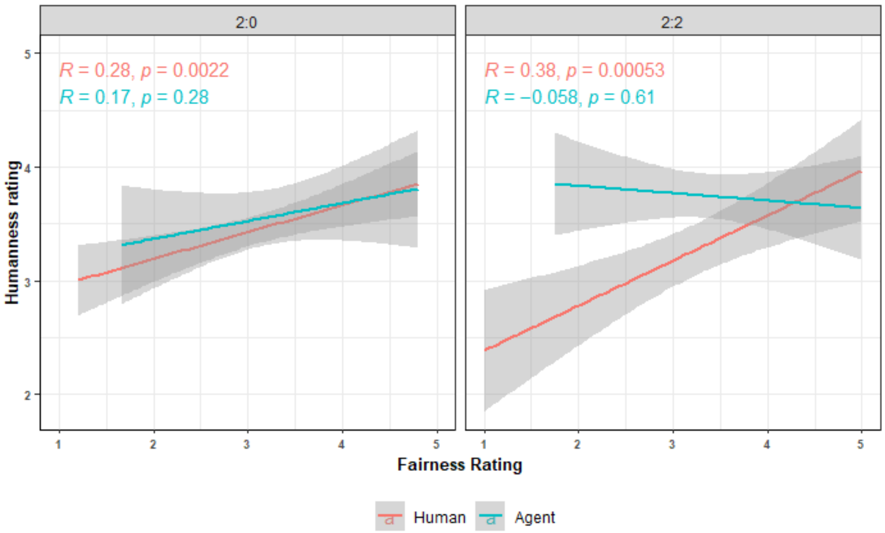

Fig. S1. Correlation between humanness and fairness ratings (Study 2)
